# Editorial: Antimicrobial nanostructured polymeric materials and nanocomposites, volume II

**DOI:** 10.3389/fbioe.2022.1015485

**Published:** 2022-09-08

**Authors:** Magdalena M. Stevanovic, Marija Vukomanovic, Marina Milenkovic, Aldo R. Boccaccini

**Affiliations:** ^1^ Group for Biomedical Engineering and Nanobiotechnology, Institute of Technical Sciences of the Serbian Academy of Sciences and Arts, Belgrade, Serbia; ^2^ Advanced Materials Department, Jožef Stefan Institute, Ljubljana, Slovenia; ^3^ Department of Microbiology and Immunology, Faculty of Pharmacy, University of Belgrade, Belgrade, Serbia; ^4^ Institute of Biomaterials, Department of Materials Science and Engineering, University of Erlangen-Nuremberg, Erlangen, Germany

**Keywords:** nanostructured materials with antimicrobial activity, nanocomposites, mechanisms of action of antimicrobial nanomaterials, nanoparticles, antimicrobial polymeric materials

## Introduction

Antimicrobial resistance (AMR) and infections caused by multidrug-resistant microbial pathogens represent one of the major clinical challenges responsible for high-level morbidity and mortality. They are a significant problem to the public health and the economic stability of societies all over the world. In November 2021 WHO has declared AMR as one of the top 10 global public health threats (Antimicrobial resistance (who.int)). According to CDC’s 2019 AMR Report, although declining since 2013, with 2.8 million new cases and more than 35,000 deaths each year, the number of people facing the AMR problem in United States is still too high (Antibiotic Resistance Threats in the United States, 2019 (cdc.gov)). AMR also remains the major health concern of EU with more than 670,000 new cases of infections caused by antibiotic-resistant bacterial strains and more than 33,000 deaths per year (Antimicrobial resistance surveillance in Europe 2022 - 2020 data (europa.eu)). In 2019 China recorded 39-% drop of antibiotic use in hospitalised patients compare to 2011 (Antimicrobial resistance - China (who.int)). Still, with 73,000 estimated new cases only for multi-drug resistant *tuberculosis*, the region remained at second position of the global highest incident rates (Antimicrobial resistance - China (who.int)). Each year a large number of people receive different kinds of implants, for example, hip or knee. However recent discoveries reveal that, either during the operative protocol or due to secondary infections, the implant’s surface could be colonized by bacteria, fungi, or both which can have serious consequences on a patient’s health. According to Annual Epidemiological Report of ECDC in 2016, post-surgical infections were identified as most common healthcare-associated infections (Surgical site infections - Annual Epidemiological Report 2016 [2014 data] (europa.eu)). In recent years it has been also recognized that microbial biofilms are ubiquitous, which has resulted in a number of studies from a biofilm perspective. Currently, great efforts are focused on the development of innovative therapeutic strategies regarding both novel drug candidates and drug delivery systems for treating microbial infections associated with implants. However, despite all these efforts as well as the urgent need, an effective and long-lasting solution to this problem is still not found. In the last decades, great attention is paid to nanostructured polymeric materials and nanocomposites because of their unique properties, which make them appropriate candidates for various applications in different medical and pharmaceutical fields. This Research Topic draws attention to the up-to-date findings regarding these issues and advanced therapeutic strategies and approaches as possible solutions.

## Description of the Research Topic

This Research Topic is focusing on progress that has been made regarding understanding the bacterial interactions, mechanisms of biofilm formation, and their persistence, as well as nanomaterials as antimicrobials, and novel alternative compounds or strategies. Implant infections are significantly increased in high-risk patients, such as populations with diabetes and immunosuppressed diseases. *Staphylococcus aureus* (*S. aureus*) is commonly recognized as the key pathogen responsible for the early failure of implants due to its ability to attach to different surfaces. Hu et al. studied the contribution of the interaction between *S. aureus* and nicotine to the initiation of peri-implant infections and bone loss in a murine osteolysis model as well as their effects on osteoblasts. The authors found that nicotine and *S. aureus* can synergistically induce peri-implant infections and concluded that targeting the interaction between nicotine and *S. aureus* can be a practical way to reduce peri-implant infections, especially in smokers.

The significance of biofilms and the process of biofilm formation as well as some of the latest research studies involving biofilm elimination have been summarized by Hong et al. The authors concluded that nanomaterials are promising delivery vehicles that can themselves act as antimicrobial agents, whose ability to penetrate biofilms holds promise as a treatment for particularly hard-to-treat infections. In the study of Miao et al. it has been demonstrated that antibacterial peptide HHC-36 as sustained-release coating of titanium dioxide nanotubes maintains effective drug release for 15 days *in vitro*, and shows significant antibacterial activity against *S. aureus*. The nano-silver loaded poly (vinyl alcohol)/keratin hydrogels which exhibited good light-permeability, mechanical strength, and antibacterial activity against *S. aureus* and *Escherichia coli* (*E. coli*) have been described in the study of Pan et al. The good antibacterial activity against *E. coli* and *S. aureus* also exhibited multilayered nanofibrous scaffolds synthesized by Liu et al. The scaffolds containing poly (l-lactic acid), polycaprolactone, and poly (vinyl alcohol) were fabricated using the electrospinning method, and the morphological, thermal, mechanical, hydrophilic, biodegradable, and release properties were analyzed. Pekarkova et al. examined the combining and the synergistic effect of selenium nanoparticles and microstructured parylene-C . The authors fabricated the parylene micropillars by oxygen plasma etching of parylene-C to introduce and study the antimicrobial effect and biocompatibility of microstructured parylene-C. Besides bacterial infections, very serious problems represent infections caused by fungi. In the review paper, Ntow-Boahen et al. provide an overview of antimicrobial polymers and nanocomposites with antifungal activity and the current understanding of their antifungal mechanisms. The authors consider that some classes of antimicrobial polymers show excellent antifungal activity, are cost-effective to synthesize, are easily chemically modified, and are also stable. In addition, they can overcome the limitations of traditional antifungal drugs as well as antimicrobial peptides. Also, antifungal polymers can be combined with other antimicrobial compounds, can be developed into nanoparticles, and incorporated into a range of nanocomposite materials to enhance antifungal activity.

## Conclusion

This Research Topic on antimicrobial nanostructured polymeric materials and nanocomposites reports up to date results on this topic obtained by different methods, encompassing not only the synthesis, characterization, and applications but also opening a window for improving existing technologies and for the development of new promising solutions. It brings together experts from different fields who have contributed cutting-edge results on this topic.

